# Impact of Daily Preventive Zinc or Therapeutic Zinc Supplementation for Diarrhea on Plasma Biomarkers of Environmental Enteric Dysfunction among Rural Laotian Children: A Randomized Controlled Trial

**DOI:** 10.4269/ajtmh.19-0584

**Published:** 2019-12-30

**Authors:** K. Ryan Wessells, Guy-Marino Hinnouho, Maxwell A. Barffour, Charles D. Arnold, Sengchanh Kounnavong, Chidchamai Kewcharoenwong, Ganjana Lertmemongkolchai, Gertrud U. Schuster, Charles B. Stephensen, Sonja Y. Hess

**Affiliations:** 1 Department of Nutrition, Institute for Global Nutrition, University of California, Davis, Davis, California;; 2 Public Health Program, College of Health and Human Services, Missouri State University, Springfield, Missouri;; 3 Lao Tropical and Public Health Institute, Vientiane, Lao People’s Democratic Republic;; 4 Mekong Health Science Research Institute, Khon Kaen University, Khon Kaen, Thailand;; 5 Faculty of Associated Medical Sciences, The Centre for Research and Development of Medical Diagnostic Laboratories, Khon Kaen University, Khon Kaen, Thailand;; 6 Agricultural Research Service, Western Human Nutrition Research Center, US Department of Agriculture, Davis, California

## Abstract

Environmental enteric dysfunction (EED) may be ameliorated by zinc supplementation. The objective of this study was to investigate the impact of different forms of zinc supplementation on biomarkers of EED (i.e., plasma citrulline, kynurenine, and tryptophan concentrations and the kynurenine:tryptophan [KT] ratio) among young Laotian children. In a double-blind randomized controlled trial, 3,407 children aged 6–23 months were randomized into one of four groups: daily preventive zinc dispersible tablets (PZ; 7 mg zinc), daily multiple micronutrient powders (MNP; 10 mg zinc, 6 mg iron, and 13 other micronutrients), therapeutic zinc supplements for diarrhea treatment (TZ; 20 mg/day for 10 days), or daily placebo powder, and followed up for ∼36 weeks. Plasma samples at baseline and endline for 359 children were analyzed for citrulline, kynurenine, and tryptophan concentrations. At baseline, the prevalence of stunting and zinc deficiency was 37% and 76.5%, respectively. The mean plasma citrulline, kynurenine, and tryptophan concentrations were 24.6 ± 5.4 µmol/L, 3.27 ± 0.83 µmol/L, and 72.3 ± 12.9 µmol/L, respectively; the mean KT ratio (×1,000) was 45.9 ± 12.0. At endline, neither plasma citrulline, kynurenine, or tryptophan concentrations, nor the KT ratio differed among intervention groups (*P* > 0.05). In this population, PZ, MNP, and TZ had no overall effect on plasma concentrations of citrulline, kynurenine, and tryptophan, or the KT ratio. The need remains to better understand the etiology of EED, and the development of biomarkers to diagnose EED and evaluate the impact of interventions.

## INTRODUCTION

Environmental enteric dysfunction (EED) is a subclinical disorder of unknown etiology, hypothesized to result from chronic exposure to inadequate sanitation and hygiene and nutrient deficiencies, and is widely prevalent among infants and young children in low- and middle-income countries.^1,2^ EED is characterized by changes in the morphology of the small intestine, including villous atrophy and crypt hyperplasia, leading to microbial translocation and intestinal and systemic inflammation.^3–5^ Resulting impairments in the absorption of nutrients and chronic immune activation may be an underlying cause of poor linear growth acquisition.^4,6^

Daily preventive zinc supplementation has been shown to increase linear growth and weight gain, reduce the incidence of diarrhea, and lower all-cause mortality among infants and young children in populations at risk for zinc deficiency.^7–9^ Therapeutic zinc supplementation, administered during episodes of diarrhea, has been shown to decrease the duration of the illness.^10^ It is possible that the effects of zinc supplementation on growth and morbidity are partially mediated by improvements in enteric function; several studies have indicated that zinc supplementation can reduce paracellular permeability and increase intestinal absorptive surface area, as measured by the dual-sugar absorption test.^11–14^ Although multiple micronutrient (MMN) supplements, in the form of MMN powders (MNP), have consistently been shown to have a beneficial impact on anemia and iron status,^15^ they have not demonstrated the same positive effects on linear growth and morbidity as PZ supplements,^16^ nor have they demonstrated improvements in intestinal permeability or absorption among infants and young children.^17–19^ In fact, some studies have indicated that MNP interventions may be associated with an increased risk of morbidity, intestinal inflammation, and altered gut microbiota.^20,21^

Given that EED is a complex disorder of unknown etiology, with no clear diagnostic criteria, a wide range of biomarkers have been evaluated to measure different aspects of the hypothesized EED pathway. A recent review by Harper et al.^4^ organized the potential biomarkers into five domains: intestinal damage and repair, permeability and absorption, microbial translocation, intestinal inflammation, and systemic inflammation. Two emerging biomarkers include plasma citrulline, a marker of intestinal damage and repair, and the kynurenine:tryptophan (KT) ratio, a marker of systemic inflammation. Citrulline is a nonessential amino acid, produced primarily by enterocytes, and can be used to assess enterocyte mass, intestinal epithelial cell loss, and absorptive function.^22^ The KT ratio is a novel biomarker of systemic immune response. During inflammation, the indoleamine 2,3-dioxygenase (IDO1) enzyme is upregulated by pro-inflammatory cytokines, such as tumor necrosis factor alpha, and tryptophan catabolism to kynurenine is increased, resulting in low tryptophan and high kynurenine concentrations and an elevated KT ratio.^23^ Tryptophan availability for protein synthesis is decreased, and low tryptophan concentrations have been associated with decreased linear growth velocity.^24,25^ Kynurenine itself can also have immunomodulatory activities, including decreasing proliferation and survival of T lymphocytes.^26^ However, evidence is inconsistent for the relationships among the different EED domains, and between each domain and stunting.^4^ In addition, few randomized controlled trials have evaluated the impact of nutritional interventions on these biomarkers of EED.

The primary objectives of the present study were to 1) compare the effects of daily preventive zinc supplementation, provided as either a single nutrient dispersible tablet or a MNP, or therapeutic zinc supplementation for diarrhea on citrulline and the tryptophan–kynurenine pathway in young Laotian children; 2) examine associations between baseline plasma citrulline, kynurenine, and tryptophan concentrations, and the KT ratio and subsequent linear growth; and 3) examine associations between baseline concentrations of citrulline, kynurenine, and tryptophan and the KT ratio and concurrent factors.

## MATERIALS AND METHODS

### Study design and participants.

The current analyses are based on data from a subsample of participants enrolled in a community-based, randomized, double-blind, placebo controlled intervention trial designed to compare the effects of a 36-week intervention providing daily preventive zinc supplementation, therapeutic zinc treatment, or a placebo control, and the effects are considered secondary outcomes. The primary outcomes were the overall effect of the interventions on 1) physical growth (length and weight), 2) incidence of diarrhea, 3) hemoglobin and micronutrient status, and 4) innate and adaptive immune response. The overall study design, data collection methods, and effect of the intervention on primary outcomes have been reported in detail elsewhere.^27,28^

In brief, the study was conducted among 3,407 infants in rural villages of Khammouane Province, central Lao PDR, from September 2015 to April 2017. Children were eligible to participate in the study if they were 6–23 months of age at enrollment, and their family planned to reside in the study catchment area for the duration of the study and was willing to accept weekly home visits. Children were excluded from the study if they presented with any of the following: severe anemia (hemoglobin < 70 g/L), severe wasting (weight-for-height *Z* score < −3 SD),^29^ bipedal edema, or other serious medical condition. In addition, children were ineligible to participate if they were currently consuming zinc supplements or currently participating in another research study.

The study was approved by the National Ethics Committee for Health Research, Ministry of Health, Lao PDR (040/2014, 069/2015, and 039/2016); the Institutional Review Board of the University of California, Davis (626187); and the Khon Kaen University, Thailand Ethics Committee on Health Research (HE572312), and registered as a clinical trial (www.ClinicalTrials.gov; NCT02428647). Consent materials were presented, both written (Lao language) and orally, in the presence of an impartial witness. Informed consent was obtained from a parent or guardian of each child before his/her enrollment in the study and documented as either a written signature or a fingerprint.

### Sample size estimation.

In the present analyses, the main outcome variables were endline plasma citrulline, kynurenine, and tryptophan concentrations and the KT ratio. To detect treatment-related differences between any two intervention groups with an effect size of 0.5 SD, a sample size of 89 participants per study intervention group was necessary (α = 0.05, β = 0.20). Given the limited literature on the effect of micronutrient supplementation on these aforementioned biomarkers, this sample size was informed by the estimated effect size of the intervention on other biomarkers, such as plasma zinc and ferritin and fecal calprotectin.^7,21,30^ The biochemistry subgroup of the parent trial (*n* = 140 per group) was a convenience sample based on logistical feasibility (i.e., all children enrolled from September to December 2015 in the two health districts closest to the study office were included in the subgroup until the sample size was met because of daily requirements for transportation of blood samples to Thailand).^27^ Samples for the present analyses were randomly selected from this subgroup, provided they met the following criteria: plasma samples available from baseline and endline collections and data available for C-reactive protein (CRP), α-1-acid glycoprotein (AGP), and hair cortisol concentrations. Fecal markers of intestinal inflammation (myeloperoxidase, neopterin, and calprotectin) were assessed in a separate subsample of study participants, enrolled from February to August 2016 because of the timing and allocation of research funding, and are thus not included in the present analyses.^27^

### Randomization and intervention products.

Eligible study participants were individually randomized to one of four intervention groups and assigned to receive the following: 1) daily PZ supplement tablets, containing 7 mg zinc and placebo tablets for diarrhea (PZ group); 2) daily preventive MNP supplements, containing 10 mg zinc, 6 mg iron, and 13 other micronutrients, and placebo tablets for diarrhea (MNP group); 3) daily placebo preventive supplements and therapeutic zinc tablets, containing 20 mg zinc for 10 days for diarrhea treatment (TZ group); and 4) daily placebo preventive powder and placebo tablets for diarrhea (control group). The tested daily PZ tablet contained 7 mg elemental zinc, based on results from a dose–response trial in which a similar dose provided the maximal benefit on diarrheal disease reduction.^31^ The tested MNP formulation contained a higher amount of zinc (10 mg zinc as zinc gluconate) and a lower amount of iron (6 mg iron as ferrous fumarate) than standard MNP formulations to address the concerns of a lack of impact on zinc status and zinc-related functional outcomes and potential adverse effects of MNP containing higher doses of iron on diarrhea.^27^ In addition, the MNP contained 0.56 mg copper, 17 μg selenium, 90 μg iodine, 400 μg RE vitamin A, 5 μg vitamin D, 5 mg vitamin E, 30 mg ascorbic acid, 0.5 mg thiamin, 0.5 mg riboflavin, 6 mg niacin, 0.5 mg vitamin B-6, 0.9 μg vitamin B-12, and 150 μg folic acid. The preventive and therapeutic zinc tablets, and the placebo tablets, were dispersible and produced by Nutriset SAS (Malaunay, France). The MNP and placebo powder were produced by DSM Fortitech Asia Pacific Sdn Bhd (Banting, Malaysia). All children, irrespective of the intervention group, received low-osmolarity oral rehydration salts for diarrhea management. Detailed information regarding supplement composition, caregiver instructions, distribution, and monitoring of adherence has been published previously.^27,28^

### Data collection.

#### Questionnaires.

Information on maternal and household socioeconomic and demographic characteristics, infant and young child feeding (IYCF) practices (breastfeeding, formula feeding, and 24-hours and 7-days food frequency questionnaire), and hygiene and sanitation practices were collected via a structured interview at baseline.^32^ Principal component analysis was used to derive a socioeconomic status index based on available indicators of household socioeconomic status, education, income, and ownership of assets, land, and animals.^28,33^ WHO indicators of adequate dietary diversity and minimum meal frequency were calculated from IYCF data.^34^ Household food insecurity was assessed using the Household Food Insecurity Access Scale (HFIAS).^35^

#### Anthropometry.

Children’s recumbent length, weight, and mid-upper arm circumference were measured at baseline, midpoint (16–20 weeks), and endline (32–36 weeks). In addition, maternal anthropometric data were collected at one time point during the intervention, with preference to baseline. All anthropometric measurements were conducted in duplicate and followed the protocols established by the Food and Nutrition Technical Assistance Project.^36^ For children, weight-for-age, length-for-age (LAZ), and weight-for-length *Z*-scores were calculated according to the WHO growth standards.^29^

### Biological samples collection.

At baseline and endline (32–36 weeks), capillary hemoglobin concentration was measured with a HemoCue^®^ Hb 301 photometer (HemoCue AB, Angelholm, Sweden). Venous blood samples (9 mL) were collected into trace element-free lithium heparin and EDTA polyethylene vacutainer tubes (Sarstedt AG & Co., Numbrecht, Germany), according to procedures recommended by the International Zinc Nutrition Consultative Group.^37^

### Morbidity surveillance and supplement administration.

For the duration of the intervention, all children were visited weekly in their homes by a morbidity surveillance worker, who was responsible for replenishing supplements, assessing adherence, and conducting a systematic symptom-based recall to ascertain the following information: general health status, appetite, stool number and consistency, presence of vomiting, nasal discharge, cough, respiratory difficulty, rapid respiration, and reported fever.

### Biological samples analysis.

Biomarkers of nutritional (plasma zinc concentration [PZC], ferritin, soluble transferrin receptor, retinol-binding protein [RBP]) and inflammatory (CRP and AGP) status were analyzed by inductively coupled plasma optical emission spectrophotometry and combined sandwich ELISA technique, as previously described.^28,38,39^ Plasma zinc concentration, ferritin, and RBP concentrations were adjusted for elevated acute-phase proteins, using a regression approach adapted from the Biomarkers Reflecting Inflammation and Nutritional Determinants of Anemia (BRINDA) project.^28,40^ Hair cortisol concentrations were measured using a commercially available salivary cortisol kit (Salimetrics, State College, PA) validated for use with human hair samples.^41,42^ Complete blood counts with automated differential counts were measured using an automated hematology analyzer (XT-1800i; Sysmex Corporation, Kobe, Japan or BC-3000Plus, Mindray Medical International Ltd., Shenzen, China). T-cell subsets (including memory cluster of differentiation [CD] 4 and CD8 T cells and regulatory T [Treg] cells) were measured from whole blood by flow cytometry (FACSCalibur; BD Biosciences, San Jose, CA). Cytokine production in the supernatants of heparinized whole blood cultures (maintained at 37°C in 5% CO_2_) was measured 24–48 hours following stimulation of the innate and adaptive immune systems (via bacterial lipopolysaccharides and anti-CD3 plus anti-CD28); cytokine concentrations from the negative control cultures are used in the present analyses.^27^ Cytokines were assayed using an electrochemiluminescence-based detection platform with multiplexed immunoassays using the U-PLEX system from MSD following the manufacturer’s protocol (Meso Scale Discovery LLC, Rockville, MD).

Analyses of citrulline, kynurenine, and tryptophan were conducted by the West Coast Metabolomics Center (University of California, Davis, CA). Lipid extracts were prepared from 20 µL plasma following the protocols first published by Matyash et al.^43^ Extracts were evaporated to dryness, reconstituted in 100 µL (80:20, v/v) acetonitrile:water (containing mixtures of internal standard), and sonicated. Following centrifugation, the supernatants were transferred to glass amber vials with micro-inserts for analysis. Human plasma from BioIVT (Westbury, NY) was used for quality control samples and extracted as described earlier. Stock solutions (1 mg/mL) of l-citrulline, l-tryptophan, and l-kynurenine were prepared individually in 80:20 v/v acetonitrile:water with labeled standards to match the resuspension procedure of the samples. Serial dilutions were completed to achieve a seven-point calibration set ranging from 0.01 to 10 µg/mL; the resulting curve was used to quantify l-citrulline, l-kynurenine, and l-tryptophan in the samples. Samples were analyzed using an 1290 Agilent HPLC (Agilent Technologies, Santa Clara, CA) coupled to the TripleTOF 6600 system (AB SCIEX, Framingham, MA). Chromatographic separation was performed on a Waters Acquity BEH Amide column (1.7 µm, 2.1 × 150 mm) at 40°C and a flow rate of 0.4 mL/minute. The mobile phases consisted of 100% of LC/MS grade water in 0.125% formic acid and 10 mM ammonium formate (A) and (95:5, v/v) acetonitrile:LC/MS grade water in 0.125% formic acid and 10 mM ammonium formate (B); the sample injection volume was 5 µL. Gradient transitions are detailed in Supplemental Table 1. MS/MS spectra were acquired according to an information-dependent acquisition; the parameters of the electrospray ionization source operating in negative mode are detailed in Supplemental Table 2.

### Statistical analyses.

A detailed statistical analysis plan was developed before analysis and is available online.^44^ All analyses were completed using a complete-case intention-to-treat approach.^45^ Descriptive statistics were calculated for all variables. Model assumptions were assessed (e.g., with Shapiro–Wilk tests for normality and assessments of linearity) and variables were appropriately transformed before subsequent analysis. MS-DIAL open-source software was used for untargeted polar metabolite analysis.^46^ Raw data were subsequently normalized by using systematic error removal using random forest (SERRF) to reduce the impact of between-series drifts of instrument sensitivity.^47^

To evaluate the impact of the intervention (objective 1), differences in mean citrulline, kynurenine, and tryptophan concentrations and the KT ratio after the intervention were assessed by ANCOVA, and dichotomous outcomes were assessed using prevalence ratios estimated with modified Poisson regression. Minimally adjusted models controlled for baseline value of the dependent variable, age at enrollment, and health district. The same methods were repeated in fully adjusted ANCOVA models, where prespecified covariates significantly associated with an outcome at 10% level of significance in a bivariate analysis were included in the final adjusted model. Prespecified effect modifiers were assessed with an interaction term in the ANCOVA; significant interactions (*P* < 0.1) are reported and further examined with stratified analyses to understand the nature of the interaction.

To examine associations with subsequent linear growth (objective 2), the predictive power of baseline citrulline, kynurenine, and tryptophan concentration and the KT ratio on subsequent change in LAZ (baseline to midline and baseline to endline) was examined, with linear regression controlling for LAZ at enrollment, age at enrollment, health district, and intervention arm. It was possible the association between the biomarker and subsequent growth would differ by intervention group, and so the interaction between baseline biomarker and intervention arm was tested before completing the analysis. To examine the relations between biomarkers of EED at baseline and associated factors (objective 3), potential factors associated with citrulline, kynurenine, and tryptophan concentrations and the KT ratio at baseline were examined with linear regression; analyses were adjusted for age at enrollment and health district.

Data were analyzed in R version 3.5.0. Data are presented as means ± SE (SD) or median (IQR), unless otherwise noted. Treatment groups remained blinded until all primary analyses were completed.

## RESULTS

### Participant characteristics.

**Figure 1.**
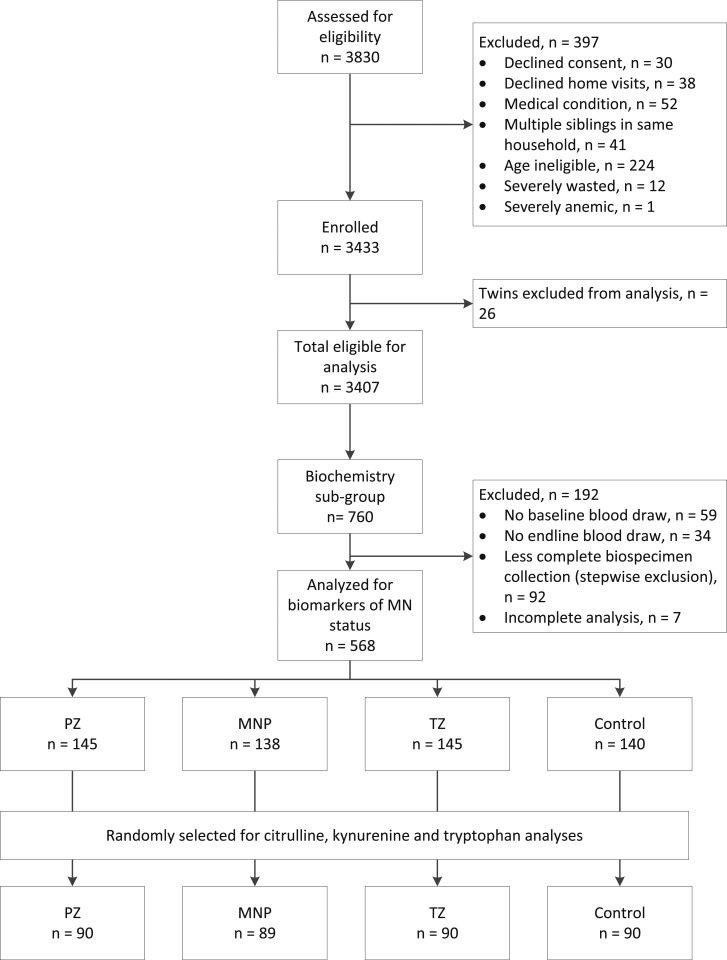
Flowchart of participant progression through the randomized controlled trial.

A total of 359 venous blood samples were analyzed for citrulline, kynurenine, and tryptophan concentrations (*n* = 89 in the MNP group and *n* = 90 in the other 3 groups; Figure 1). The mean age at enrollment was 16.0 ± 4.9 months. The prevalence of stunting and zinc deficiency was high, at 37.0% and 76.5%, respectively (Table 1). Children in the control arm of the intervention generally had lower prevalence of stunting (27.8% versus ∼40%), underweight (15.6% versus ∼32%), and wasting (2% versus ∼8%) than those in the other three intervention arms. One-quarter of children had elevated CRP and/or AGP (> 5 mg/L and > 1 g/L, respectively), indicative of systemic subclinical inflammation. Two-thirds of families had consistent latrine access and 85% obtained drinking water from an improved source as defined by the WHO,^48^ but only one-quarter of caregivers reported always practicing handwashing with soap after defecation and/or before meal preparation.

**Table 1:** Child, maternal, and household characteristics of the study participants at baseline by intervention group

Variables	All	PZ	MNP	TZ	Control
N*	359	90	89	90	90
Age (months)†	16.0 ± 4.9	15.8 ± 5.1	16.7 ± 4.7	16.1 ± 4.7	15.7 ± 5.1
District					
Nongbok	242 (67.4)	61 (67.8)	63 (70.8)	62 (68.9)	56 (62.2)
Xebangfai	117 (32.6)	29 (32.2)	26 (29.2)	28 (31.1)	34 (37.8)
Males, *n* (%)	157 (43.7)	34 (37.8)	46 (51.7)	38 (42.2)	39 (43.3)
Anthropometry					
LAZ	−1.7 ± 1.1	−1.8 ± 1.1	−1.9 ± 1.1	−1.9 ± 0.9	−1.3 ± 1.1
WAZ	−1.4 ± 1.0	−1.5 ± 1.1	−1.5 ± 1.0	−1.5 ± 1.0	−1.1 ± 1.0
WLZ	−0.7 ± 0.9	−0.8 ± 0.9	−0.8 ± 0.8	−0.8 ± 0.9	−0.5 ± 0.9
Stunted, *n* (%)	133 (37.0)	35 (38.9)	35 (39.3)	38 (42.2)	25 (27.8)
Underweight, *n* (%)	100 (27.9)	29 (32.2)	27 (30.3)	30 (33.3)	14 (15.6)
Wasted, *n* (%)	25 (7.0)	9 (10.0)	8 (9.0)	6 (6.7)	2 (2.2)
Biochemical indicators‡					
Citrulline (µmol/L)	24.6 ± 5.4	24.6 ± 5.5	24.9 ± 4.5	24.2 ± 5.8	24.8 ± 5.7
Kynurenine (µmol/L)	3.3 ± 0.8	3.4 ± 0.8	3.1 ± 0.8	3.3 ± 0.9	3.2 ± 0.8
Tryptophan (µmol/L)	72.3 ± 12.9	73.6 ± 12.2	73.7 ± 14.1	71.0 ± 13.0	71.1 ± 12.2
KT ratio (ratio × 1,000)	45.9 ± 12.0	46.6 ± 11.7	43.3 ± 9.7	47.3 ± 12.5	46.5 ± 13.2
Hemoglobin (Hb) (g/L)	110.7 ± 9.1	108.8 ± 9.4	112.0 ± 9.3	110.9 ± 8.7	111.3 ± 8.7
Hb < 110 g/L	155 (43.2)	42 (46.7)	38 (42.7)	40 (44.4)	35 (38.9)
CRP (mg/L)	0.47 (0.22, 1.88)	0.70 (0.23, 2.31)	0.39 (0.23, 1.00)	0.46 (0.20, 2.59)	0.38 (0.20, 2.04)
CRP > 5 mg/L	44 (12.3)	15 (16.7)	5 (5.6)	13 (14.4)	11 (12.2)
AGP (g/L)	0.61 (0.45, 0.90)	0.66 (0.44, 1.07)	0.62 (0.45, 0.85)	0.55 (0.49, 0.90)	0.60 (0.44, 0.86)
AGP > 1 g/L	78 (21.7)	24 (26.7)	16 (18.0)	19 (21.1)	19 (21.1)
PZC (µg/dL)	57.4 ± 12.1	56.7 ± 13.7	58.3 ± 11.1	57.8 ± 10.7	57.0 ± 12.7
PZC < 65 µg/dL	274 (76.5)	67 (74.4)	65 (73.0)	69 (77.5)	73 (81.1)
Retinol-binding protein concentration (µmol/L)	1.3 ± 0.3	1.2 ± 0.3	1.3 ± 0.3	1.3 ± 0.3	1.3 ± 0.3
Ferritin (µg/L)	20.7 (11.7, 31.9)	21.3 (11.9, 36.1)	20.2 (10.7, 28.7)	19.1 (12.4, 29.9)	22.7 (12.8, 35.7)
Storage iron deficiency (ferritin < 12 µg/L)	92 (25.6)	23 (25.6)	26 (29.2)	22 (24.4)	21 (23.3)
sTfR (mg/L)	9.4 (7.7, 15.1)	9.4 (7.1, 15.8)	9.3 (7.6, 15.1)	9.5 (7.7, 13.1)	9.4 (7.8, 16.3)
Functional iron deficiency (sTfR > 8.3 mg/L)	237 (66.0)	57 (63.3)	58 (65.2)	61 (67.8)	61 (67.8)
Hair cortisol (pg/mg)	21.6 (13.9, 34.9)	27.9 (15.0, 39.8)	17.2 (12.6, 26.2)	21.0 (13.0, 34.4)	21.3 (15.3, 32.0)
Infant and young child feeding practices§					
Breastfed, in the previous month, *n* (%)	207 (64.7)	50 (66.7)	46 (59.7)	55 (67.1)	56 (65.1)
ADD, *n* (%)	117 (36.3)	26 (34.7)	28 (35.9)	25 (30.1)	38 (44.2)
MMF, *n* (%)	69 (21.6)	15 (20.0)	16 (20.8)	17 (20.7)	21 (24.4)
Maternal characteristics					
Maternal age (years)	26.7 ± 6.1	26.5 ± 6.0	27.7 ± 6.8	26.3 ± 6.2	26.3 ± 5.4
Maternal BMI (kg/m^2^)	21.9 ± 3.3	21.9 ± 3.4	21.9 ± 2.8	21.7 ± 3.2	22.2 ± 3.6
Maternal education (completed primary school), *n* (%)	165 (47.0)	35 (40.7)	43 (48.3)	42 (48.3)	44 (50.6)
Household characteristics§					
HFIAS (moderately or severely food insecure), *n* (%)	166 (46.9)	41 (46.6)	36 (41.9)	49 (54.4)	40 (44.4)
Latrine access, *n* (%)	204 (63.4)	49 (65.3)	51 (65.4)	49 (59.0)	55 (64.0)
Handwashing, *n* (%)	78 (24.2)	19 (25.3)	15 (19.2)	23 (27.7)	21 (24.4)
Use of improved drinking water source, *n* (%)	239 (84.5)	54 (78.3)	61 (84.7)	60 (88.2)	64 (86.5)
Animals in household, *n* (%)	238 (78.8)	59 (80.8)	58 (77.3)	60 (82.2)	61 (75.3)

ADD = adequate dietary diversity; AGP = α_1_-acid glycoprotein; BMI = body mass index; CRP = C-reactive protein; HFIAS = household food insecurity access scale; KT = kynurenine:tryptophan; LAZ = length-for-age *Z*-score; MMF = minimum meal frequency; MNP = micronutrient powder; PZ = preventive zinc; PZC = plasma zinc concentration; sTfR = soluble transferrin receptor; TZ = therapeutic zinc; WAZ = weight-for-age *Z*-score; WLZ = weight-for-length *Z*-score.

* Values presented as mean ± SD, median (IQR), or *n* (%).

† Breastfeeding, MMF, *n* = 320; ADD, latrine access, *n* = 322; maternal BMI, *n* = 281; improved drinking water source, *n* = 283; animals in household, *n* = 302.

‡ Plasma zinc, retinol-binding protein, and ferritin concentrations adjusted for elevated acute-phase proteins based on Barffour et al.,^28^ and estimates of the prevalence of deficiency are based on inflammation-adjusted micronutrient biomarker concentrations.

§ Infant and young child feeding practices defined by the WHO^32^; HFIAS from the Food and Nutrition Technical Assistance III Project^35^; handwashing defined as report of consistent handwashing after defecation and/or before meal preparation compared with occasional or no handwashing after defecation and before meal preparation; improved drinking water source defined by the WHO.^48^

At baseline, the mean plasma citrulline concentration was 24.6 ± 5.4 µmol/L (Table 1). The mean plasma kynurenine and tryptophan concentrations and the KT ratio (×1000) were 3.3 ± 0.8 µmol/L, 72.3 ± 12.9 µmol/L, and 45.9 ± 12.0, respectively. Few children (5.3%) had low plasma citrulline concentrations (< 17 µmol/L), and no children had low tryptophan concentrations (< 35 µmol/L). Baseline citrulline, kynurenine, and tryptophan concentrations were positively correlated with one another; the KT ratio correlated negatively with citrulline and tryptophan, and positively with kynurenine (Table 2).

**Table 2:** Partial Spearman correlation coefficient for environmental enteric dysfunction biomarkers at baseline, adjusted for age at enrollment and district

	Citrulline	Kynurenine	Tryptophan	KT ratio
Citrulline	–	0.182†	0.427‡	−0.109*
Kynurenine		–	0.389‡	0.711‡
Tryptophan			–	−0.302‡
KT ratio				–

KT = kynurenine:tryptophan. *P < 0.05, †*P* < 0.01, ‡*P* < 0.001.

### Impact of the intervention.

In both minimally adjusted (adjusted for baseline value of outcome of interest, age, and health district) and fully adjusted models, endline concentrations of citrulline, kynurenine, and tryptophan and the KT ratio did not differ among the four groups (Table 3). In the minimally adjusted models, children who received therapeutic zinc for the treatment of diarrhea (TZ) were more likely to have kynurenine concentrations in the highest tertile of study participants, and marginally less likely to have tryptophan concentrations in the lowest tertile, than the other three treatment groups (*P* = 0.035 and *P* = 0.079, respectively; Table 3). There were no between-group tertile differences in the KT ratio or plasma citrulline concentrations. Baseline citrulline concentration modified the effect of the intervention on final citrulline concentrations (*P* for interaction = 0.075; data not shown). Among children with the highest plasma citrulline concentrations at baseline, there was a ∼10 µmol/L greater endline citrulline concentration among children who received the placebo compared with the PZ intervention. However, there were no between-group differences in endline citrulline concentration among children in the lower two tertiles of citrulline concentrations at baseline.

**Table 3:** Effects of 32–40 weeks of supplementation with daily preventive zinc, daily multiple micronutrient powder, or therapeutic zinc for diarrhea on endline citrulline, kynurenine, and tryptophan concentrations and the KT ratio among young Laotian children

Outcome	PZ	MNP	TZ	Control	Minimally adjusted *P*-value*	Adjusted *P*-value*
Citrulline (µmol/L)†	24.9 (23.7, 26.1)	25.8 (24.6, 27.0)	26.3 (25.1, 27.5)	26.4 (25.2, 27.6)	0.287	0.359
Kynurenine (µmol/L)	3.1 (2.9, 3.2)	3.0 (2.9, 3.2)	3.2 (3.1, 3.4)	3.0 (2.8, 3.2)	0.115	0.156
Tryptophan (µmol/L)	66.7 (63.8, 69.6)	66.0 (63.1, 68.9)	70.3 (67.4, 73.2)	66.4 (63.5, 69.4)	0.151	0.273
KT ratio (ratio ×1,000)	47.0 (44.6, 49.4)	46.5 (44.0, 48.9)	46.6 (44.2, 49.0)	46.4 (44.1, 48.8)	0.988	0.988
Low tertile citrulline (%)	41.7 (31.5, 51.9)	31.8 (21.9, 41.7)	32.0 (22.9, 41.0)	29.4 (20.4, 38.4)	0.276	0.347
High tertile kynurenine (%)	31.7 (23.0, 40.3)	26.8 (17.6, 35.9)	44.5 (34.6, 54.4)	29.1 (20.0, 38.1)	0.035	0.070
Low tertile tryptophan (%)	34.7 (24.8, 44.7)	41.3 (31.3, 51.2)	23.6 (15.4, 31.8)	34.5 (25.1, 43.9)	0.079	0.069
High tertile KT ratio (%)	33.1 (23.5, 42.8)	37.4 (27.3, 47.5)	29.9 (20.6, 39.2)	32.5 (23.1, 41.9)	0.705	0.460

KT = kynurenine:tryptophan; MNP = micronutrient powder; PZ = preventive zinc; TZ = therapeutic zinc.

* Minimally adjusted and fully adjusted models control for age and district of enrollment. Fully adjusted models included variables significantly associated (*P* < 0.1) with the respective outcome. Considered covariates were child characteristics of gender, enrollment anthropometry, infant and young feeding practices, and concentrations of α_1_-acid glycoprotein, C-reactive protein, soluble transferrin receptor, retinol-binding protein, ferritin, hair cortisol, and plasma zinc; maternal characteristics of age, education, and BMI; and household characteristics of household food insecurity, asset index, sanitation and hygiene measures, and month of enrollment.

† Values represent estimate (means) and 95% CI for continuous outcomes, and percentage and 95% CI for dichotomous outcomes.

### Associations with subsequent linear growth.

Baseline plasma citrulline, kynurenine, and tryptophan concentrations and the KT ratio were not associated with subsequent linear growth, measured 16–20 weeks and 32–40 weeks following biomarker assessment (Table 4).

**Table 4:** Associations between baseline plasma citrulline, tryptophan, and kynurenine concentrations, and the kynurenine–tryptophan ratio and changes in LAZ over time among young Laotian children

Baseline variable	Change in LAZ 16–20 weeks after baseline biomarker measure*	*P*-value	Change in LAZ 32–40 weeks after baseline biomarker measure*	*P*-value
Citrulline (µmol/L)	0.000 (−0.006, 0.006)	0.88	0.004 (−0.003, 0.011)	0.26
Kynurenine (µmol/L)	0.017 (−0.022, 0.055)	0.40	0.015 (−0.03, 0.06)	0.51
Tryptophan (µmol/L)	0.000 (−0.003, 0.002)	0.87	0.000 (−0.003, 0.002)	0.81
KT ratio (ratio*1,000)	0.001 (−0.001, 0.004)	0.32	0.001 (−0.002, 0.004)	0.42

KT = kynurenine:tryptophan; LAZ = length-for-age *Z* score.

* Models adjusted for baseline value of outcome of interest, treatment group, age at enrollment, and district.

### Biomarkers of EED at baseline and associated predictors.

With increasing child age, citrulline concentrations increased, and kynurenine and the KT ratio decreased (Table 5). Kynurenine was negatively associated with LAZ at baseline; however, there were no other associations between markers of intestinal injury repair and systemic inflammation and anthropometric indicators of obtained growth. Concentrations of citrulline and tryptophan decreased, and the KT ratio increased, with increasing concentrations of acute-phase proteins (CRP and AGP). Citrulline was positively associated with PZC and RBP concentrations, and tryptophan was positively associated with RBP concentration. There were no observed relationships with IYCF practices, maternal characteristics, or food security. However, citrulline and tryptophan were positively associated with socioeconomic status, and tryptophan and kynurenine concentrations were lower among children in households with no animals. Citrulline concentrations were higher among children in households using an improved drinking water source, but lower in households practicing handwashing.

**Table 5:** Concurrent factors associated with citrulline, kynurenine, and tryptophan concentrations and the KT ratio at baseline in young Laotian children

Variables	Citrulline (µmol/L)§	Kynurenine (µmol/L)	Tryptophan (µmol/L)	KT ratio (ratio × 1,000)
Age (months)	0.14 (0.03, 025)*	−0.03 (−0.05, −0.01)†	−0.21 (−0.48, 0.07)	−0.30 (−0.55, −0.05)*
District				
Nongbok	Ref	Ref	Ref	Ref
Xebangfai	0.05 (−1.14, 1.23)	−0.16 (−0.35, 0.02)	−1.54 (−4.38, 1.31)	−1.03 (−3.67, 1.61)
Gender				
Male	Ref	Ref	Ref	Ref
Female	0.32 (−0.8, 1.44)	0.01 (−016, 0.18)	−0.20 (−2.89, 2.50)	0.44 (−2.05, 2.92)
Anthropometry				
LAZ	0.02 (−0.51, 0.55)	−0.09 (−0.17, −0.01)*	−0.67 (−1.94, 0.61)	−0.85 (−2.03, 0.33)
WAZ	−0.07 (−0.63, 0.48)	−0.7 (−2.03, 0.63)	−0.05 (−0.14, 0.03)	−0.28 (−1.51, 0.95)
WLZ	−0.14 (−0.77, 0.49)	0.0 (−0.1, 0.1)	−0.52 (−2.04, 1.00)	0.45 (−0.96, 1.85)
Stunting (LAZ < −2 SD)	0.12 (−1.05, 1.28)	0.17 (−0.01, 0.34)	0.74 (−2.07, 3.55)	2.02 (−0.56, 4.61)
Underweight (WAZ < −2 SD)	0.14 (−1.11, 1.39)	0.13 (−0.06, 0.32)	2.03 (−0.98, 5.04)	0.41 (−2.37, 3.19)
Wasting (WLZ < −2 SD)	0.37 (−1.81, 2.55)	−0.08 (−0.41, 0.25)	−0.22 (−5.48, 5.04)	−0.88 (−5.73, 3.97)
Biochemical indicators‖				
Hemoglobin (Hb)	0.38 (−0.25, 1.00)	0.03 (−0.06, 0.13)	0.57 (−0.94, 2.08)	0.01 (−1.38, 1.41)
Anemia (Hb < 110 g/L)	−0.40 (−1.53, 0.73)	0.01 (−0.16, 0.18)	−0.70 (−3.43, 2.02)	0.73 (−1.79, 3.25)
CRP (mg/L)	−1.02 (−1.37, −0.67)‡	0.0 (−0.05, 0.06)	−1.80 (−2.66, −0.93)‡	1.11 (0.3, 1.91)†
CRP ≥ 5 mg/L	−3.65 (−5.30, −2.00)‡	−0.24 (−0.5, 0.01)	−6.41 (−10.44, −2.38)†	0.13 (3.63, 3.90)
AGP (g/L)	−2.80 (−3.78, −1.82)‡	0.05 (−0.11, 0.21)	−3.17 (−5.62, −0.72)*	2.84 (0.58, 5.10)*
AGP ≥ 1 g/L	−3.12 (−4.43, −1.81)‡	−0.01 (−0.22, 0.20)	−3.88 (−7.11, −0.64)*	2.39 (−0.61, 5.39)
PZC (µg/dL)	3.01 (0.32, 5.69)*	−0.01 (−0.42, 0.4)	4.72 (−1.79, 11.2)	−3.79 (−9.80, 2.22)
PZC ≤ 65 µg/dL	−1.09 (−2.39, 0.22)	0.13 (−0.07, 0.33)	−0.35 (−3.50, 2.80)	2.32 (−0.58, 5.22)
Retinol-binding protein concentration (µmol/L)	4.17 (1.52, 6.81)†	0.14 (−0.27, 0.55)	7.42 (0.99, 13.85)*	−3.46 (−9.42, 2.51)
Ferritin (µg/L)	−0.37 (−1.10, 0.36)	0.01 (−0.11, 0.12)	−1.25 (−3.02, 0.52)	0.85 (−0.79, 2.48)
Storage iron deficiency (ferritin < 12 µg/L)	0.82 (−0.45, 2.09)	−0.02 (−0.22, 0.17)	1.76 (−1.30, 4.82)	−1.24 (−4.07, 1.58)
sTfR (mg/L)	−0.56 (−1.85, 0.73)	0.02 (−0.18, 0.21)	0.11 (−3.01, 3.22)	0.28 (−2.59, 3.15)
Functional iron deficiency (sTfR > 8.3 mg/L)	−0.50 (−1.68, 0.68)	0.14 (−0.04, 0.32)	−0.88 (−3.73, 1.97)	2.75 (0.14, 5.37)*
Hair cortisol (pg/mg)	−0.08 (−0.72, 0.56)	−0.04 (−0.14, 0.06)	−1.84 (−3.37, −0.31)*	0.54 (−0.88, 1.96)
Infant and young child feeding practices¶#				
Breastfed, in the previous month	−0.75 (−2.1, 0.59)	−0.16 (−0.36, 0.04)	−2.67 (−4.91, 1.57)	−1.47 (−4.39, 1.44)
Adequate dietary diversity	0.69 (−0.55, 1.93)	−0.02 (−0.20, 0.17)	2.68 (−0.31, 5.67)	−2.06 (−4.78, 0.67)
Minimum meal frequency	0.17 (−1.09, 1.43)	−0.05 (−0.23, 0.14)	−0.48 (−3.52, 2.56)	−0.53 (−3.27, 2.20)
Maternal characteristics¶				
Maternal age (years)	−0.01 (−0.10, 0.08)	0.0 (−0.01, 0.02)	0.17 (−0.06, 0.40)	−0.08 (−0.29, 0.13)
Maternal BMI (kg/m^2^)	−0.07 (−0.27, 0.12)	−0.01 (−0.04, 0.02)	−0.08 (−0.54, 0.38)	−0.16 (−0.59, 0.27)
Maternal education (years)	−0.08 (−0.28, 0.12)	0.0 (−0.03, 0.03)	−0.1 (−0.58, 0.39)	−0.02 (−0.47, 0.43)
Household characteristics¶#				
HFIAS	−0.04 (−0.2, 0.12)	0.0 (−0.02, 0.03)	−0.25 (−0.64, 0.14)	0.19 (−0.16, 0.55)
Socioeconomic status index	0.38 (0.08, 0.68)*	0.0 (−0.05, 0.05)	0.87 (0.15, 1.60)*	−0.50 (−1.16, 0.17)
No animals in household	−0.27 (−1.73, 1.20)	−0.29 (−0.51, −0.06)*	−5.45 (−9.04, −1.87)†	−0.65 (−3.98, 2.67)
Use of improved drinking water source	1.75 (0.04, 3.46)*	0.09 (−0.17, 0.35)	3.45 (−0.83, 7.72)	−1.36 (−5.18, 2.46)
Handwashing	−1.64 (−3.04, −0.25)*	0.05 (−0.16, 0.26)	−1.03 (−4.43, 2.37)	1.34 (−1.75, 4.44)
Latrine access	0.31 (−0.95, 1.58)	0.06 (−0.13, 0.25)	1.1 (−1.96, 4.16)	0.45 (−2.34, 3.24)

AGP = α_1_-acid glycoprotein; BMI = body mass index; CRP = C-reactive protein; HFIAS = household food insecurity access scale; KT = kynurenine:tryptophan; LAZ = length-for-age *Z*-score; PZ = preventive zinc; PZC = plasma zinc concentration; sTfR = soluble transferrin receptor; TZ = therapeutic zinc; WAZ = weight-for-age *Z*-score; WLZ = weight-for-length *Z*-score. *P < 0.05, †*P* < 0.01, ‡*P* < 0.001.

§ Models adjusted for age at enrollment and district. Values represent beta coefficient and 95% CI from linear regression models. For continuous predictors, this represents the change in outcome corresponding to a one unit increase in the predictor. For dichotomous predictors, this corresponds to the change in mean concentration from the reference group.

‖ AGP, CRP, PZC, retinol-binding protein, ferritin, sTfR, and hair cortisol were natural log-transformed; PZC, retinol-binding protein, and ferritin were adjusted for elevated acute-phase proteins^28^; and estimates of the prevalence of deficiency are based on inflammation-adjusted micronutrient biomarker concentrations.

¶ Breastfeeding, MMF, *n* = 320; ADD, latrine access, *n* = 322; maternal BMI, *n* = 281; improved drinking water source, *n* = 283; animals in household, *n* = 302.

# Infant and young child feeding practices defined by the WHO^32^; HFIAS from the Food and Nutrition Technical Assistance III Project^35^; socioeconomic status index based on available indicators of household socioeconomic status, education, income, and ownership of assets, land, and animals; handwashing defined as report of consistent handwashing after defecation and/or before meal preparation compared with occasional or no handwashing after defecation and before meal preparation; improved drinking water source defined by the WHO.^48^

The KT ratio was not associated with blood leukocyte concentrations (lymphocytes, monocytes, neutrophils, eosinophils, and basophils) (Table 6). However, all T-lymphocyte measures (total and memory CD4^+^ and CD8^+^ T cells, as well as CD4^+^ Treg cells) were negatively associated with the KT ratio, save memory CD8 T cells, which showed a positive association. These relationships were primarily driven by statistically significant relationships between tryptophan (rather than kynurenine) and the immune marker of interest, expect for memory CD8 T cells, where the opposite was true. The KT ratio was also compared with cytokine production by unstimulated leukocytes cultured ex vivo, which should reflect the level of activation of these cells at the time of the blood collection. In this analysis, the KT ratio was positively associated with interleukin (IL)-10 and IL-6 concentrations.

**Table 6:** Concurrent predictors associated with citrulline, kynurenine, and tryptophan concentrations and the KT ratio at baseline in young Laotian children: immune biomarkers

Variables	*N*	Citrulline (µmol/L)§	Kynurenine (µmol/L)	Tryptophan (µmol/L)	KT (ratio × 1,000)
Leukocytes in peripheral blood (cells/µL)					
Neutrophils	324	−0.74 (−1.86, 0.39)	−0.11 (−0.28, 0.07)	0.07 (−2.77, 2.91)	−1.96 (−4.56, 0.64)
Eosinophils	324	0.28 (−0.1, 0.66)	−0.01 (−0.07, 0.05)	0.49 (−0.47, 1.45)	−0.44 (−1.32, 0.44)
Basophils‖	157	0.01 (−0.13, 0.16)	0.01 (−0.13, 0.16)	0.33 (−1.89, 2.54)	−0.19 (−2.31, 1.94)
Lymphocytes	324	0.05 (−0.19, 0.28)	0.03 (−0.01, 0.06)	0.89 (0.32, 1.46)†	−0.17 (−0.7, 0.37)
Monocytes	323	−0.66 (−1.37, 0.05)	−0.06 (−0.17, 0.05)	−0.9 (−2.68, 0.88)	−0.39 (−2.03, 1.24)
T-cell subsets in peripheral blood (cells/mL)					
CD4 total	271	1.00 (0.00, 2.01)*	−0.15 (−0.31, 0.00)	4.04 (1.53, 6.55)†	−4.72 (−6.97, −2.48)‡
CD4 Treg total	271	8.93 (−2.87, 20.73)	−1.47 (−3.27, 0.33)	40.16 (10.81, 69.52)†	−48.71 (75.17, −22.26)‡
CD8 total	271	1.01 (−1.38, 3.40)	−0.03 (−0.40, 0.34)	8.41 (2.43, 14.38)†	−5.56 (−11.02, −0.10)*
CD4 memory	271	5.30 (−0.64, 11.23)	−0.71 (−1.63, 0.20)	10.32 (−4.72, 25.36)	−16.72 (−30.28, −3.16)*
CD8 memory	271	−2.31 (−10.82, 6.2)	1.61 (0.31, 2.90)*	−1.57 (−23.11, 19.96)	27.94 (8.67, 47.2) †
Cytokines produced by cultured whole blood cells with LPS stimulation (negative control cultures) (pg/mL)					
IL-10	264	0.17 (−0.25, 0.58)	0.01 (−0.05, 0.08)	−1.18 (−2.23, −0.14)*	1.04 (0.09, 1.99)*
IL-6	264	0.00 (−0.28, 0.28)	0.01 (−0.03, 0.06)	−0.78 (−1.48, −0.08)*	0.65 (0.01, 1.29)*
IL-1β	264	0.03 (−0.32, 0.37)	0.02 (−0.03, 0.08)	−0.72 (−1.6, 0.15)	0.77 (−0.02, 1.57)
TNFα	264	0.10 (−0.35, 0.55)	0.01 (−0.06, 0.08)	−0.71 (−1.87, 0.44)	0.59 (−0.46, 1.64)
Cytokines produced by cultured whole blood cells with T-cell stimulation (negative control cultures) (pg/mL)					
IFN-γ	267	−0.17 (−0.44, 0.10)	0.03 (−0.02, 0.07)	−0.02 (−0.71, 0.68)	0.48 (−0.15, 1.11)
IL-2	267	0.21 (−0.04, 0.46)	−0.01 (−0.05, 0.03)	0.15 (−0.49, 0.79)	−0.26 (−0.84, 0.32)
IL-13	267	0.08 (−0.22, 0.38)	−0.05 (−0.10, −0.01)*	−0.33 (−1.09, 0.42)	−0.49 (−1.18, 0.2)
IL-17	267	−0.10 (−0.44, 0.23)	0.00 (−0.06, 0.05)	−0.66 (−1.52, 0.2)	0.36 (−0.43, 1.15)

CD = cluster of differentiation; IFN = interferon; IL = interleukin; KT = kynurenine:tryptophan; TNF = tumor necrosis factor; Treg = regulatory T cells. *P < 0.05, †*P* < 0.01, ‡*P* < 0.001.

§ Models adjusted for age at enrollment and district. Values represent beta coefficient and 95% CI from linear regression models. For continuous predictors, this represents the change in outcome corresponding to a one unit increase in the predictor.

‖ Children with basophils below the limit of detection are not included in this analysis (basophil concentration is included as a continuous variable). When basophil concentration was treated as a categorical variable (above or below the limit of detection), the results were consistent.

## DISCUSSION

### Overview of main results.

In the present study, we found that neither preventive nor therapeutic zinc supplementation, nor the provision of MNP, had an effect on concentrations of plasma citrulline, kynurenine, or tryptophan or the KT ratio when provided to children 6–24 months of age daily for 9 months. In addition, plasma concentrations of these EED biomarkers at baseline were not predictive of subsequent linear growth, measured ∼4.5 and 9 months after initial biomarker assessment.

### Impact of the intervention.

The present study is the first randomized controlled trial that examined the impact of supplementation with zinc or MNP on the KT ratio, as a potential novel indicator of changes in systemic inflammation and EED, making comparisons to the existing body of literature difficult. Similarly, few studies have investigated the effects of MMN supplementation on plasma citrulline concentration, as described in more detail later. It is possible that the lack of effect of zinc and MMN supplementation on these biomarkers of EED in the present study results from one or more of the following: 1) the intervention had no impact on EED (either because the intervention was ineffective or the population was not sufficiently at risk), or 2) the measured biomarkers are nonsensitive indicators of EED, and/or there is a possible domain-specific response of EED to zinc supplementation (e.g., impacts limited to improvements in intestinal permeability or absorptive surface area). These aforementioned possibilities are examined in further detail in the following.

First, it is possible the micronutrient intervention had no impact on EED. In the parent trial, PZ supplements and MNP reduced the prevalence of zinc deficiency, and MNP reduced the prevalence of iron deficiency, indicating that the population was responsive to nutritional supplementation.^28^ However, there were no overall treatment effects on linear growth or morbidity.^28,49^ In addition, there was no overall impact on markers of intestinal inflammation (neopterin, myeloperoxidase, and calprotectin) among children participating in a separate sub-study of the parent trial,^50^ also suggesting that the intervention may not have affected EED. However, age did modify the impact of the intervention on diarrhea incidence and duration; exploratory analyses indicated that therapeutic zinc for the treatment of diarrhea reduced the burden of diarrhea among children aged > 18 months at the time of enrollment.^49^ By contrast, age did not modify the effect of the intervention on markers of EED. We did observe a marginally significant effect of therapeutic zinc on categorical outcomes. Namely, children who received therapeutic zinc for the treatment of diarrhea were less likely to be in the lowest tertile of tryptophan; however, they were also more likely to be in the highest tertile of kynurenine concentrations and all children had tryptophan concentrations in the “normal range” (above the cutoff for low concentrations), so findings have unlikely health or programmatic implications. Thus, the measured biomarkers of EED may not be on the impact pathway between intervention (therapeutic zinc) and outcome (reduction in diarrhea incidence and burden), or the sample size for the present analyses may have been too small to detect age-related differences in impact. It is also possible that there is a low prevalence of EED in the population, and thus, the population did not have the potential to respond to the intervention. In the present study, 5% of children had low citrulline concentrations at baseline and no children had low plasma tryptophan concentrations.^51^ However, given the suggested high prevalence of EED globally,^52^ it is likely that some children may have had EED, particularly considering the elevated prevalence of stunting and systemic inflammation (elevated CRP and/or AGP), low adequate dietary diversity, and limited access to and uptake of adequate sanitation and hygiene facilities and practices. Indeed, in the fecal markers of intestinal inflammation sub-study of the present parent trial, > 50% of children had elevated myeloperoxidase, neopterin, and calprotectin concentrations compared with standards from adults and children living in nontropical regions (G. M. Hinnouho, personal communication).

Second, it is possible that the measured biomarkers are nonsensitive indicators of EED, and/or there is a possible domain-specific response of EED to zinc supplementation. Only one previous study investigated the effect of short-term zinc supplementation on plasma citrulline concentrations among young children in Burkina Faso and found no effect.^53^ By contrast, several studies have demonstrated that zinc can reduce paracellular permeability and/or increase intestinal absorptive surface area, as assessed using a dual-sugar absorption test,^11–14^ although other trials which included MMN supplements have not.^17–19^ However, there is limited information available from histopathological analyses suggesting that MMN supplementation may increase villous height and absorptive area among adults with EED.^54^ Thus, it is possible that one or more of the interventions (PZ, TZ, or MNP) improved aspects of the EED domain of permeability and absorption, but these did not translate to effects on biomarkers of intestinal damage and repair, as measured by citrulline concentration, or systemic inflammation as assessed by the KT ratio.^4^

### Prediction of subsequent linear growth.

The recent systematic review by Harper et al.^4^ found conflicting evidence of associations between different EED domains and linear growth. Although there is strong evidence to support the relationship between intestinal inflammation and poor linear growth,^55–58^ the evidence of association between intestinal damage and repair and stunting, and between systemic inflammation and stunting is inconsistent. In the present study, baseline concentrations of citrulline, kynurenine, and tryptophan and the KT ratio were not associated with a subsequent 4.5- or 9-month change in LAZ among infants and young children aged 6–24 months. Similarly, in the MAL-ED longitudinal cohort study which examined relationships between the aforementioned biomarkers and statural growth failure, Kosek et al. also found no significant relationships between citrulline, kynurenine, and the KT ratio and subsequent linear growth among infants and young children aged > 6 months in Peru and Tanzania.^24^ Similar results were observed in the Brazil cohort for markers of systemic inflammation (kynurenine and the KT ratio), although investigators did note that higher citrulline concentrations were predictive of less stunting among female children.^58^ However, plasma tryptophan concentrations were positively associated with subsequent linear growth among all children in Peru and Tanzania and among boys only in Brazil,^24,58^ which is supported by evidence from animal studies indicating negative effects of tryptophan deficiency on growth velocity,^25^ but is in contrast to our null results. Tryptophan may not have been a growth-limiting nutrient in this population considering that mean tryptophan concentrations were 72.3 ± 12.9 µmol/L at baseline, and no children had low plasma tryptophan concentrations (< 34 µmol/L).^51^ In comparison, mean (95% CI) tryptophan concentrations in the MAL-ED Peru and Tanzania cohorts were 49.9 (22.1–74.9) µmol/L. Kosek et al. postulated that these low tryptophan concentrations, possibly due to low dietary tryptophan intake, alterations in the intestinal microbiome, or increased IDO1 activity due to inflammation, and subsequent modifications in tryptophan metabolism led to the observed linear growth deficits.^24,59^ In a subsample of 720 participants from the current parent study, among which EED was assessed by markers of intestinal inflammation, fecal myeloperoxidase concentrations, but not neopterin or calprotectin concentrations, were associated with subsequent acquisition of linear growth deficits in the 4 months following the initial biomarker assessment.^50^

### Biomarkers at baseline and associated factors.

Concurrent factors consistently associated with citrulline, kynurenine, and tryptophan concentrations and the KT ratio tended to cluster into three main categories: 1) age, 2) indicators of systemic inflammation and immune function, and 3) measures of sanitation and hygiene. There was some indication that citrulline and tryptophan were positively associated with household wealth (e.g., socioeconomic status index and the presence of animals in a household). However, indicators of anthropometry, IYCF practices, and maternal characteristics had limited significant associations with citrulline, kynurenine, and tryptophan concentrations and the KT ratio, which is supported by some,^24,60^ but not all,^24,58^ of the previous literature.

In the present study, citrulline was negatively associated with concurrent concentrations of acute-phase proteins (CRP and AGP) and the KT ratio, and was positively associated with kynurenine and tryptophan concentrations, providing further evidence for the relationship between intestinal damage and repair and systemic inflammation.^4^ However, we observed no significant associations between cell-signaling cytokines and citrulline concentrations. The KT ratio was positively associated with concurrent concentrations of acute-phase proteins (CRP and AGP) and interleukins (IL-6 and IL-10), driven by significant negative associations between markers of systemic inflammation and tryptophan concentrations, similar to those observed by Kosek et al.^24^ However, somewhat unexpectedly, we did not observe a significant association between interferon-γ (IFN-γ), a pro-inflammatory cytokine which stimulates IDO1 production,^61^ and tryptophan, kynurenine, or the KT ratio. However, we may have seen the expected association if we had measured plasma IFN-γ, as was reported previously,^24^ rather that ex vivo production of IFN-γ by unstimulated leukocytes in culture. Decreased tryptophan and increased kynurenine concentrations, and increased KT ratios, have been reported during infections in animal models,^25^ likely due to increased IDO1 activity during inflammation, which may serve to deprive pathogens of essential amino acids (i.e., tryptophan) necessary for growth and proliferation, and regulate T-cell activity, leading to immunological tolerance (i.e., decreases in tryptophan decrease T-cell proliferation at sites of inflammation, leading to decreased activity of T cells).^62^ The negative association of the KT ratio with T-cell concentrations observed in the present study was not entirely unexpected as higher plasma tryptophan could well be associated with better maintenance of T-cell homeostasis in healthy individuals, although such data have not been reported previously, to the best of our knowledge. The positive association of the KT ratio with memory CD8 T cells was unexpected but could represent a positive association of kynurenine production during undiagnosed infections in some of our study participants (e.g., viral infections) that would cause an expansion of activated CD8 T cells (which would be CD45RO^+^) in blood. There is a precedent for such an association in HIV infection, where a positive association of the KT ratio with total CD8 T cells has been reported,^63^ as well as with activated CD8 T cells.^63,64^ Confirmation of this hypothesis would require further work with more specific characterization of T-cell subsets.

In the present study, there were limited associations between indicators of water, sanitation, and hygiene (WSH), and concentrations of citrulline, kynurenine, and tryptophan and the KT ratio. Three recent community-based randomized controlled effectiveness trials of WSH interventions, designed on the premise that EED is caused by fecal–oral contamination in conditions of poor quality WSH, have recently been completed.^65–68^ In Bangladesh, WSH interventions lowered intestinal permeability and inflammation in children aged 3 months compared with controls, but were subsequently associated with higher biomarkers at 28 months of age, possibly because of an intervention-related delay in peak EED.^69^ However, in all three trials, the WSH interventions failed to impact child growth, indicating the interventions may not have been intensive enough to impact the contributions of EED to childhood stunting, or the pathway between EED biomarkers and stunting is weak.^65–68^

### Strengths and weaknesses.

The randomized placebo-controlled study design and rigorous data collection lend strength to these findings. In addition, the study was implemented in a population with a high prevalence of zinc deficiency and stunting,^28^ and thus was conducted in a population with a presumably high prevalence of EED and potential to respond to the interventions. A primary limitation of this study is the lack of clear diagnostic criteria for EED. Previous studies have shown positive impacts of zinc supplementation on lactulose: mannitol ratios,^1^ reflective of intestinal permeability and absorptive capacity, and the EED biomarker most consistently affected by zinc supplementation; however, this biomarker was not measured in the current study. In addition, although we analyzed fecal markers of intestinal inflammation among a subgroup of participants in the same parent trial, we were unable to measure citrulline, kynurenine, and tryptophan concentrations in the same participants.^50^ Thus, we are limited in our comparison of the performance of the KT ratio as a candidate biomarker for assessing EED to other more established biomarkers, and cannot contribute to the evidence base for the relationships among the different EED domains. Nevertheless, the present study provided new insights into the lack of impact of MNP and zinc supplementation on the measured indicators.

## CONCLUSION

In the present study, preventive and therapeutic zinc supplementation, or a MMN powder, did not have an impact on concentrations of citrulline, kynurenine, and tryptophan and the KT ratio. In addition, citrulline and the KT ratio were not predictors of subsequent linear growth acquisition. The need remains to better understand the etiology of EED, and the development of biomarkers to diagnose EED and evaluate the impact of interventions.

## Supplemental file

Supplemental tables

## References

[1] Lindenmayer GW Stoltzfus RJ Prendergast AJ, 2014 Interactions between zinc deficiency and environmental enteropathy in developing countries. Adv Nutr 5: 1–6. 24425714 10.3945/an.113.004838 PMC3884090

[2] Sanitation Hygiene Infant Nutrition Efficacy Trial Team, Humphrey JH 2015 The Sanitation Hygiene Infant Nutrition Efficacy (SHINE) trial: rationale, design, and methods. Clin Infect Dis 61 (Suppl 7): S685–S702. 26602296 10.1093/cid/civ844 PMC4657589

[3] Lindenbaum J Harmon JW Gerson CD, 1972 Subclinical malabsorption in developing countries. Am J Clin Nutr 25: 1056–1061. 4562265 10.1093/ajcn/25.10.1056

[4] Harper KM Mutasa M Prendergast AJ Humphrey J Manges AR, 2018 Environmental enteric dysfunction pathways and child stunting: a systematic review. PLoS Negl Trop Dis 12: e0006205. 29351288 10.1371/journal.pntd.0006205 PMC5792022

[5] Crane RJ Jones KD Berkley JA, 2015 Environmental enteric dysfunction: an overview. Food Nutr Bull 36 (Suppl 1): S76–S87. 25902619 10.1177/15648265150361S113 PMC4472379

[6] Oria RB Murray-Kolb LE Scharf RJ Pendergast LL Lang DR Kolling GL Guerrant RL, 2016 Early-life enteric infections: relation between chronic systemic inflammation and poor cognition in children. Nutr Rev 74: 374–386. 27142301 10.1093/nutrit/nuw008 PMC4892302

[7] Brown KH Peerson JM Baker SK Hess SY, 2009 Preventive zinc supplementation among infants, preschoolers, and older prepubertal children. Food Nutr Bull 30 (Suppl 1): S12–S40. 19472600 10.1177/15648265090301S103

[8] Mayo-Wilson E Junior JA Imdad A Dean S Chan XH Chan ES Jaswal A Bhutta ZA, 2014 Zinc supplementation for preventing mortality, morbidity, and growth failure in children aged 6 months to 12 years of age. Cochrane Database Syst Rev 5: 1 CD009384. 10.1002/14651858.CD009384.pub2 24826920

[9] Liu E Pimpin L Shulkin M Kranz S Duggan CP Mozaffarian D Fawzi WW, 2018 Effect of zinc supplementation on growth outcomes in children under 5 years of age. Nutrients 10: E377. 29558383 10.3390/nu10030377 PMC5872795

[10] Lazzerini M Ronfani L, 2013 Oral zinc for treating diarrhoea in children. Cochrane Database Syst Rev 12: CD005436. 10.1002/14651858.CD005436.pub4 23440801

[11] Bates CJ 1993 A trial of zinc supplementation in young rural Gambian children. Br J Nutr 69: 243–255. 8457531 10.1079/bjn19930026

[12] Roy SK Behrens RH Haider R Akramuzzaman SM Mahalanabis D Wahed MA Tomkins AM, 1992 Impact of zinc supplementation on intestinal permeability in Bangladeshi children with acute diarrhoea and persistent diarrhoea syndrome. J Pediatr Gastroenterol Nutr 15: 289–296. 1432467 10.1097/00005176-199210000-00010

[13] Alam AN Sarker SA Wahed MA Khatun M Rahaman MM, 1994 Enteric protein loss and intestinal permeability changes in children during acute shigellosis and after recovery: effect of zinc supplementation. Gut 35: 1707–1711. 7829006 10.1136/gut.35.12.1707 PMC1375257

[14] Ryan KN Stephenson KB Trehan I Shulman RJ Thakwalakwa C Murray E Maleta K Manary MJ, 2014 Zinc or albendazole attenuates the progression of environmental enteropathy: a randomized controlled trial. Clin Gastroenterol Hepatol 12: 1507.e1–1513.e1. 24462483 10.1016/j.cgh.2014.01.024

[15] De-Regil LM Suchdev PS Vist GE Walleser S Pena-Rosas JP, 2011 Home fortification of foods with multiple micronutrient powders for health and nutrition in children under two years of age. Cochrane Database Syst Rev 9: CD008959. 10.1002/14651858.CD008959.pub2 21901727

[16] Lamberti LM Fischer Walker CL Black RE, 2016 Zinc deficiency in childhood and pregnancy: evidence for intervention effects and program responses. World Rev Nutr Diet 115: 125–133. 27198901 10.1159/000442079

[17] Wang AZ Shulman RJ Crocker AH Thakwalakwa C Maleta KM Devaraj S Manary MJ Trehan I, 2017 A combined intervention of zinc, multiple micronutrients, and albendazole does not ameliorate environmental enteric dysfunction or stunting in rural Malawian children in a double-blind randomized controlled trial. J Nutr 147: 97–103. 27807040 10.3945/jn.116.237735

[18] Smith HE 2014 Multiple micronutrient supplementation transiently ameliorates environmental enteropathy in Malawian children aged 12-35 months in a randomized controlled clinical trial. J Nutr 144: 2059–2065. 25411039 10.3945/jn.114.201673

[19] Kelly P Shawa T Mwanamakondo S Soko R Smith G Barclay GR Sanderson IR, 2010 Gastric and intestinal barrier impairment in tropical enteropathy and HIV: limited impact of micronutrient supplementation during a randomised controlled trial. BMC Gastroenterol 10: 72. 20604937 10.1186/1471-230X-10-72 PMC2910659

[20] Soofi S Cousens S Iqbal SP Akhund T Khan J Ahmed I Zaidi AK Bhutta ZA, 2013 Effect of provision of daily zinc and iron with several micronutrients on growth and morbidity among young children in Pakistan: a cluster-randomised trial. Lancet 382: 29–40. 23602230 10.1016/S0140-6736(13)60437-7

[21] Jaeggi T 2015 Iron fortification adversely affects the gut microbiome, increases pathogen abundance and induces intestinal inflammation in Kenyan infants. Gut 64: 731–742. 25143342 10.1136/gutjnl-2014-307720

[22] Crenn P Vahedi K Lavergne-Slove A Cynober L Matuchansky C Messing B, 2003 Plasma citrulline: a marker of enterocyte mass in villous atrophy-associated small bowel disease. Gastroenterology 124: 1210–1219. 12730862 10.1016/s0016-5085(03)00170-7

[23] DeBoer MD 2018 Early Life Interventions for Childhood Growth and Development in Tanzania (ELICIT): a protocol for a randomised factorial, double-blind, placebo-controlled trial of azithromycin, nitazoxanide and nicotinamide. BMJ Open 8: e021817. 10.1136/bmjopen-2018-021817 PMC6042604 29982218

[24] Kosek MN 2016 Plasma tryptophan and the kynurenine-tryptophan ratio are associated with the acquisition of statural growth deficits and oral vaccine underperformance in populations with environmental enteropathy. Am J Trop Med Hyg 95: 928–937. 27503512 10.4269/ajtmh.16-0037 PMC5062803

[25] Le Floc’h N Otten W Merlot E, 2011 Tryptophan metabolism, from nutrition to potential therapeutic applications. Amino Acids 41: 1195–1205. 20872026 10.1007/s00726-010-0752-7

[26] Cervenka I Agudelo LZ Ruas JL, 2017 Kynurenines: tryptophan’s metabolites in exercise, inflammation, and mental health. Science 357: eaaf9794. 28751584 10.1126/science.aaf9794

[27] Wessells KR 2018 Comparison of two forms of daily preventive zinc supplementation versus therapeutic zinc supplementation for diarrhea on young children's physical growth and risk of infection: study design and rationale for a randomized controlled trial. BMC Nutr 4: 39. 10.1186/s40795-018-0247-6 PMC7050875 32153900

[28] Barffour MA 2019 Effects of daily zinc, daily multiple micronutrient powder, or therapeutic zinc supplementation for diarrhea prevention on physical growth, anemia, and micronutrient status in rural Laotian children: a randomized controlled trial. J Pediatr 207: 80.e2–89.e2. 30580974 10.1016/j.jpeds.2018.11.022 PMC6448681

[29] de Onis M; World Health Organization Multicentre Growth Reference Study Group, 2007 WHO Child Growth Standards based on length/height, weight and age. Acta Paediatr 95: 76–85. 10.1111/j.1651-2227.2006.tb02378.x 16817681

[30] Adu-Afarwuah S Lartey A Brown KH Zlotkin S Briend A Dewey KG, 2008 Home fortification of complementary foods with micronutrient supplements is well accepted and has positive effects on infant iron status in Ghana. Am J Clin Nutr 87: 929–938. 18400716 10.1093/ajcn/87.4.929

[31] Wuehler SE Sempertegui F Brown KH, 2008 Dose-response trial of prophylactic zinc supplements, with or without copper, in young Ecuadorian children at risk of zinc deficiency. Am J Clin Nutr 87: 723–733. 18326612 10.1093/ajcn/87.3.723

[32] World Health Organization 2008 Indicators for Assessing Infant and Young Child Feeding Practices: Part 1 Definitions. Geneva, Switzerland: WHO.

[33] Vyas S Kumaranayake L, 2006 Constructing socio-economic status indices: how to use principal components analysis. Health Policy Plan 21: 459–468. 17030551 10.1093/heapol/czl029

[34] World Health Organization 2010 Indicators for Assessing Infant and Young Child Feeding Practices: Part 2 Measurement. Geneva, Switzerland: WHO.

[35] Coates J Swindale A Bilinksky P Household Food Insecurity Access Scale (HFIAS) for Measurement of Household Food Access: Indicator Guide (V. 3). Washington, DC: FHI 360/FANTA, 2007. Available at: https://www.fantaproject.org/monitoring-and-evaluation/household-food-insecurity-access-scale-hfias. Accessed November 7, 2013.

[36] Cogill B, 2003 Anthropometric Indicators Measurement Guide. Washington, DC: Food and Nutrition Technical Assistance Project, Academy for Educational Development.

[37] International Zinc Nutrition Consultative Group, 2012 Assessing Population Zinc Status with Serum Zinc Concentration. IZiNCG technical brief no. 2 Available at: http://www.izincg.org. Accessed March 26, 2014.

[38] Erhardt JG Estes JE Pfeiffer CM Biesalski HK Craft NE, 2004 Combined measurement of ferritin, soluble transferrin receptor, retinol binding protein, and C-reactive protein by an inexpensive, sensitive, and simple sandwich enzyme-linked immunosorbent assay technique. J Nutr 134: 3127–3132. 15514286 10.1093/jn/134.11.3127

[39] Killilea DW Ames BN, 2008 Magnesium deficiency accelerates cellular senescence in cultured human fibroblasts. Proc Natl Acad Sci USA 105: 5768–5773. 18391207 10.1073/pnas.0712401105 PMC2311331

[40] Namaste SM Aaron GJ Varadhan R Peerson JM Suchdev PS; BRINDA Working Group, 2017 Methodologic approach for the biomarkers reflecting inflammation and nutritional determinants of anemia (BRINDA) project. Am J Clin Nutr 106 (Suppl 1): 333S–347S. 28615254 10.3945/ajcn.116.142273 PMC5490643

[41] Vaghri Z Guhn M Weinberg J Grunau RE Yu W Hertzman C, 2013 Hair cortisol reflects socio-economic factors and hair zinc in preschoolers. Psychoneuroendocrinology 38: 331–340. 22809790 10.1016/j.psyneuen.2012.06.009 PMC4821190

[42] Hinnouho GM Bernstein RM Barffour MA Arnold CD Wessells KR Ratsavong K Bounheuang B Kounnavong S Hess SY, 2018 Impact of two forms of daily preventive zinc or therapeutic zinc supplementation for diarrhea on hair cortisol concentrations among rural Laotian children: a randomized controlled trial. Nutrients 11: E47. 30591656 10.3390/nu11010047 PMC6356851

[43] Matyash V Liebisch G Kurzchalia TV Shevchenko A Schwudke D, 2008 Lipid extraction by methyl-tert-butyl ether for high-throughput lipidomics. J Lipid Res 49: 1137–1146. 18281723 10.1194/jlr.D700041-JLR200 PMC2311442

[44] Hess SY Barffour MA Hinnouho GM, 2019 Lao Zinc Study. Open Science Framework. Available at: https://osf.io/5bq9c. Accessed May 13, 2019.

[45] Johnston BC Guyatt GH, 2016 Best (but oft-forgotten) practices: intention-to-treat, treatment adherence, and missing participant outcome data in the nutrition literature. Am J Clin Nutr 104: 1197–1201. 27733397 10.3945/ajcn.115.123315

[46] Tsugawa H Cajka T Kind T Ma Y Higgins B Ikeda K Kanazawa M VanderGheynst J Fiehn O Arita M, 2015 MS-DIAL: data-independent MS/MS deconvolution for comprehensive metabolome analysis. Nat Methods 12: 523–526. 25938372 10.1038/nmeth.3393 PMC4449330

[47] Fan S Kind T Cajka T Hazen SL Tang WHW Kaddurah-Daouk R Irvin MR Arnett DK Barupal DK Fiehn O, 2019 Systematic error removal using random forest for normalizing large-scale untargeted lipidomics data. Anal Chem 91: 3590–3596. 30758187 10.1021/acs.analchem.8b05592 PMC9652764

[48] World Health Organization, 2017 Safely Managed Drinking Water–Thematic Report on Drinking Water 2017. Geneva, Switzerland: World Health Organization Available at: https://washdata.org/monitoring/drinking-water. Accessed February 27, 2019.

[49] Barffour MA 2018 Effects of two forms of daily preventive zinc and therapeutic zinc supplementation for diarrhea on diarrhea and acute respiratory tract infections in Laotian children (OR32-05). Curr Dev Nutr 2: nzy039.

[50] Hinnouho GM Wessells KR Barffour M Sayasone S Arnold C Kounnavong S Brown K Hess S, 2019 Effects of different strategies for delivering supplemental zinc on selected fecal markers of environmental enteric dysfunction among young Laotian children (P04-012-19). Curr Dev Nutr 3: nzz051. 10.4269/ajtmh.20-0106 PMC7543857 32618258

[51] Lepage N McDonald N Dallaire L Lambert M, 1997 Age-specific distribution of plasma amino acid concentrations in a healthy pediatric population. Clin Chem 43: 2397–2402. 9439460

[52] Syed S Ali A Duggan C, 2016 Environmental enteric dysfunction in children. J Pediatr Gastroenterol Nutr 63: 6–14. 26974416 10.1097/MPG.0000000000001147 PMC4920693

[53] Wessells KR Hess SY Rouamba N Ouedraogo ZP Kellogg M Goto R Duggan C Ouedraogo JB Brown KH, 2013 Associations between intestinal mucosal function and changes in plasma zinc concentration following zinc supplementation. J Pediatr Gastroenterol Nutr 57: 348–355. 23689263 10.1097/MPG.0b013e31829b4e9e PMC4627695

[54] Louis-Auguste J Greenwald S Simuyandi M Soko R Banda R Kelly P, 2014 High dose multiple micronutrient supplementation improves villous morphology in environmental enteropathy without HIV enteropathy: results from a double-blind randomised placebo controlled trial in Zambian adults. BMC Gastroenterol 14: 15. 24428805 10.1186/1471-230X-14-15 PMC3897937

[55] Kosek M 2013 Fecal markers of intestinal inflammation and permeability associated with the subsequent acquisition of linear growth deficits in infants. Am J Trop Med Hyg 88: 390–396. 23185075 10.4269/ajtmh.2012.12-0549 PMC3583335

[56] Arndt MB 2016 Fecal markers of environmental enteropathy and subsequent growth in Bangladeshi children. Am J Trop Med Hyg 95: 694–701. 27352872 10.4269/ajtmh.16-0098 PMC5014281

[57] Kosek MN; Mal-Ed Network Investigators, 2017 Causal pathways from enteropathogens to environmental enteropathy: findings from the MAL-ED birth cohort study. EBioMedicine 18: 109–117. 28396264 10.1016/j.ebiom.2017.02.024 PMC5405169

[58] Guerrant RL 2016 Biomarkers of environmental enteropathy, inflammation, stunting, and impaired growth in children in northeast Brazil. PLoS One 11: e0158772. 27690129 10.1371/journal.pone.0158772 PMC5045163

[59] Takikawa O Yoshida R Kido R Hayaishi O, 1986 Tryptophan degradation in mice initiated by indoleamine 2,3-dioxygenase. J Biol Chem 261: 3648–3653. 2419335

[60] Gosselin K 2015 Serum citrulline does not predict stunting or environmental enteric dysfunction in Tanzanian and Malawian infants. FASEB J 29: 403.5.

[61] Taylor MW Feng GS, 1991 Relationship between interferon-gamma, indoleamine 2,3-dioxygenase, and tryptophan catabolism. FASEB J 5: 2516–2522. 1907934

[62] Van der Leek AP Yanishevsky Y Kozyrskyj AL, 2017 The kynurenine pathway as a novel link between allergy and the gut microbiome. Front Immunol 8: 1374. 29163472 10.3389/fimmu.2017.01374 PMC5681735

[63] Serrano-Villar S 2014 HIV-infected individuals with low CD4/CD8 ratio despite effective antiretroviral therapy exhibit altered T cell subsets, heightened CD8+ T cell activation, and increased risk of non-AIDS morbidity and mortality. PLoS Pathog 10: e1004078. 24831517 10.1371/journal.ppat.1004078 PMC4022662

[64] Gelpi M Hartling HJ Ueland PM Ullum H Troseid M Nielsen SD, 2017 Tryptophan catabolism and immune activation in primary and chronic HIV infection. BMC Infect Dis 17: 349. 28511640 10.1186/s12879-017-2456-z PMC5434617

[65] Humphrey JH 2019 Independent and combined effects of improved water, sanitation, and hygiene, and improved complementary feeding, on child stunting and anaemia in rural Zimbabwe: a cluster-randomised trial. Lancet Glob Health 7: e132–e147. 30554749 10.1016/S2214-109X(18)30374-7 PMC6293965

[66] Prendergast AJ 2019 Independent and combined effects of improved water, sanitation, and hygiene, and improved complementary feeding, on stunting and anaemia among HIV-exposed children in rural Zimbabwe: a cluster-randomised controlled trial. Lancet Child Adolesc Health 3: 77–90. 30573417 10.1016/S2352-4642(18)30340-7 PMC6472652

[67] Luby SP 2018 Effects of water quality, sanitation, handwashing, and nutritional interventions on diarrhoea and child growth in rural Bangladesh: a cluster randomised controlled trial. Lancet Glob Health 6: e302–e315. 29396217 10.1016/S2214-109X(17)30490-4 PMC5809718

[68] Null C 2018 Effects of water quality, sanitation, handwashing, and nutritional interventions on diarrhoea and child growth in rural Kenya: a cluster-randomised controlled trial. Lancet Glob Health 6: e316–e329. 29396219 10.1016/S2214-109X(18)30005-6 PMC5809717

[69] Lin A 2019 Effects of water, sanitation, handwashing, and nutritional interventions on environmental enteric dysfunction in young children: a cluster-randomized controlled trial in rural Bangladesh. Clin Infect Dis ciz291, doi:10.1093/cid/ciz291. 30963177 10.1093/cid/ciz291

