# Supplementary material for: Impact of Daily Preventive Zinc or Therapeutic Zinc Supplementation for Diarrhea on Plasma Biomarkers of Environmental Enteric Dysfunction among Rural Laotian Children: A Randomized Controlled Trial
Source: Am J Trop Med Hyg. 2019 Dec 30;102(2):415–26. doi: 10.4269/ajtmh.19-0584 (PMC7008314; doi:10.4269/ajtmh.19-0584)
Supplement: Supplementary file 1 [file tpmd190584.SD1.pdf]

Supplemental Table 1. Gradient transitions for HILIC-MS analysis

Time (minutes)	Mobile phase A[1] (%)	Mobile phase B[1] (%)
0	0	100
0 – 2	0	100
2 – 7.7	30	70
7.7 – 9.5	60	40
9.5 – 10.25	70	30
10.25 – 12.75	0	100
12.75 – 16.75	0	100

[1]Mobile phase A consisted of 100% of LC/MS grade water in 0.125% formic acid and 10 mM ammonium formate; Mobile phase B consisted of (95:5, v/v) acetonitrile: LC/MS grade water in 0.125% formic acid and 10 mM ammonium formate.

Supplemental Table 2. Parameters of the electron spray ionization (ESI) source operating in negative mode for HILIC-MS Analysis

**Parameter**	**Value**
Ion source gas 1, psi	650
Ion source gas 2, psi	50
Curtain gas, psi	35
Source temperature, °C	5000
Ion spray voltage floating, V	-4000
Declustering potential	60
Collision energy, V	10
